# Artificial intelligence for postpartum hemorrhage: a systematic review

**DOI:** 10.3389/fgwh.2026.1806220

**Published:** 2026-06-12

**Authors:** Rawan AlSaad, Farah Yazbek, Thomas Farrell, Shima Albasha, Almayasa Al-Marri, Rajat Thomas

**Affiliations:** 1 AI Center for Precision Health, Weill Cornell Medicine-Qatar, Doha, Qatar; 2 Department of Obstetrics and Gynecology, Hamad Medical Corporation, Doha, Qatar; 3 College of Medicine, Qatar University, Doha, Qatar

**Keywords:** artificial intelligence, hemorrhage, maternal, obstetrics, postpartum, pregnancy, women's health

## Abstract

**Background:**

Postpartum hemorrhage (PPH) remains a leading cause of maternal morbidity and mortality, and conventional risk tools often rely on static factors that may miss rapidly evolving intrapartum events. Artificial intelligence (AI) offers data-driven, potentially dynamic approaches for PPH prediction, yet prior syntheses have provided limited coverage of recent methods and validation practices.

**Objective:**

To systematically synthesize the evidence on AI models for PPH prediction, including clinical applications, data sources, prediction targets, validation strategies, and study quality.

**Methods:**

We conducted a PRISMA-guided systematic review of studies published between 2015 and 2025 across MEDLINE, Embase, Scopus, IEEE Xplore, and Google Scholar. Two reviewers independently screened records, extracted data using a prespecified form, and resolved disagreements by consensus. Risk of bias and applicability were appraised using a modified QUADAS-2 tool tailored to AI-based PPH prediction. Findings were synthesized narratively across study design, prediction context, inputs, modeling approaches, and validation strategies.

**Results:**

In total, 33 studies met the eligibility criteria. Publications were concentrated in 2023-2025 (79%) and were predominantly retrospective (91%) and single-site (55%). Most models targeted anticipatory risk stratification (79%), with fewer addressing intrapartum/immediate postpartum early warning or severity escalation (24%). Outcomes primarily modeled PPH occurrence (79%) as binary classification (91%). All studies used closed datasets. Classical machine learning dominated (85%), while deep learning (33%) and LLM-based approaches (6%) were less frequent. Validation was mainly internal (76%), with limited external validation. Participant selection was the main quality concern (high risk of bias: 48%), while index test and reference standard were largely low risk.

**Conclusions:**

AI-based PPH prediction research is rapidly expanding but remains constrained by retrospective, closed datasets, heterogeneous outcome definitions, and limited external/temporal validation. Progress toward clinical readiness requires harmonized labeling, multicenter datasets, deployment-oriented evaluation, and prospective implementation studies.

## 1 Introduction

Postpartum hemorrhage (PPH) is typically defined as excessive bleeding after childbirth. PPH is a leading cause of maternal mortality worldwide, affecting around 14 million women each year and resulting in about 70,000 maternal deaths (1). The World Health Organization (WHO) defines PPH as ≥500 mL of blood loss within 24 hours after birth, whereas the American College of Obstetricians and Gynecologists (ACOG) definition uses a higher threshold of ≥1,000 mL cumulative blood loss within 24 hours and considers any amount of blood loss accompanied by clinical signs of hypovolemia as PPH (2, 3). Despite advances in healthcare, PPH remains a challenge even in high-resource settings. Beyond mortality, PPH is associated with serious complications, including shock, transfusion, hysterectomy, and prolonged hospitalization (4). Earlier identification of risk (or early detection of evolving hemorrhage) can enable preparedness (e.g., blood bank activation, escalation pathways, uterotonics, and multidisciplinary readiness) and timelier treatment to reduce preventable harm (3, 5).

However, current clinical approaches for prediction of PPH, such as risk checklists, category-based tools, and clinician judgment, often rely heavily on static antenatal factors and may miss a substantial proportion of events, particularly those that evolve rapidly intrapartum or in low-risk patients (6). Validation work on widely used risk stratification approaches has shown only moderate sensitivity in some real-world cohorts, indicating that many PPH cases occur despite low-risk classification at admission (7). These limitations motivate more individualized, data-driven methods that can update risk dynamically as labor and delivery unfold (6, 8).

In parallel, artificial intelligence (AI) methods, including classical machine learning, deep learning, and emerging large language model (LLM) approaches, are increasingly applied to predict obstetric complications (9–11). In PPH specifically, studies have evaluated antenatal risk stratification, intrapartum early-warning/severity escalation, and automated case identification from documentation using features such as demographics, comorbidities, obstetric history, medications, peripartum events, hemodynamics, and derived phenotypes from notes.

Recent reviews on AI for postpartum hemorrhage risk assessment remain fragmented and limited in scope, leaving major methodological and translational gaps. In 2025, four reviews addressed PPH prediction, yet the two systematic efforts were constrained by small AI evidence bases and narrow modeling coverage: Baeta et al. (12) included only nine machine-learning studies while largely synthesizing traditional statistical models, and Sirichaisit et al. (13) analyzed 11 studies only. The remaining 2025 articles [Mathewlynn et al. (14); Wakefield et al. (15)] were narrative/primer-style and did not follow a systematic approach, limiting reproducibility and introducing potential selection bias. Earlier work was similarly restricted, with the 2023 systematic review (16) synthesizing only four studies. Overall, prior reviews provide limited coverage of recent AI approaches (e.g., deep learning and LLMs), rigorous validation, and real-world implementation considerations, and they lack comprehensive synthesis of the evidence.

To address these gaps, the current systematic review synthesizes 33 studies spanning the full spectrum of AI models for predicting PPH. Specifically, we (1) catalog PPH prediction model characteristics, (2) characterize data sources used for AI modeling, (3) summarize prediction targets, (4) synthesize model evaluation strategies, (5) assess risk of bias and applicability using a modified QUADAS-2 framework tailored to PPH prediction studies, and (6) identify gaps related to clinical deployment to inform priorities for future research and responsible implementation.

## 2 Methods

This systematic review was conducted in accordance with the Preferred Reporting Items for Systematic Reviews and Meta-Analyses (PRISMA) guidelines (17). The review protocol was not prospectively registered in PROSPERO or any other public registry. The following sections provide a detailed description of the methods used in this review.

### 2.1 Study eligibility criteria

We applied prespecified eligibility criteria using the Population–Concept–Context (PCC) framework to determine study inclusion.

#### 2.1.1 Inclusion criteria

- Population/Problem: Human studies involving pregnant or postpartum individuals in any care setting (hospital, clinic, or community), across all geographic regions and income settings.
- Concept (AI for PPH prediction): Studies that developed and/or validated artificial intelligence models intended to predict postpartum hemorrhage (PPH) risk or onset. Eligible methods included, but were not limited to classical machine-learning algorithms (e.g., logistic regression, regularized regression, random forest, support vector machine, gradient boosting, XGBoost, LightGBM, CatBoost, and k-nearest neighbors), deep-learning architectures (e.g., multilayer perceptrons, convolutional neural networks, ResNet-based models, and GAN-based approaches), and LLM/NLP-based methods. Logistic regression and regularized regression were included only when implemented as predictive algorithms within a model-development and validation framework (e.g., comparative model benchmarking, supervised prediction, hold-out/cross-validation, temporal validation, or external validation), and were not included when used solely for conventional inferential/statistical analysis, risk-factor estimation, or standalone nomogram construction.
- Context: Any healthcare or research context in which PPH prediction could be applied, including antenatal risk stratification, intrapartum early warning, immediate postpartum surveillance, and early postpartum monitoring.
- Target condition/outcomes: The primary target was PPH as defined by individual studies (e.g., quantified/estimated blood loss thresholds such as ≥500 mL or ≥1000 mL, ICD-coded PPH), severity measures (quantified blood loss or hemoglobin decline), and clinically relevant outcomes (transfusion, escalation of uterotonics/interventions, or composite hemorrhage outcomes).
- Study types, language, dates: Model development, internal validation, external validation, and model updating studies, including retrospective or prospective cohorts, registry studies, pragmatic implementations, and randomized trials (if available). English-language studies published from 2015 to present were eligible.

#### 2.1.2 Exclusion criteria

- Population/Problem: Non-human/animal studies.
- Concept: Studies not focused on predicting PPH (e.g., treatment optimization without prediction) and purely descriptive analytics without predictive modeling.
- Publication types: Editorials, commentaries, and narrative reviews.
- Reporting limitations: Abstract-only publications lacking adequate information on methods and performance.

### 2.2 Information sources and search strategy

We conducted a comprehensive literature search across five electronic databases: MEDLINE (Ovid), Embase (Ovid), Scopus, IEEE Xplore, and Google Scholar. The full database-specific search strategies, including the exact queries and retrieved record counts, are provided in Supplementary Material 1. Search queries were constructed by combining two core concepts: postpartum hemorrhage AND artificial intelligence, incorporating relevant synonyms and controlled vocabulary terms where applicable. Searches were limited to studies published between 1 January 2015 and 19 December 2025. We restricted the search to studies published from 2015 onward to focus on the contemporary period of AI-based clinical prediction research, during which modern machine-learning, deep-learning, and LLM-based approaches became more established in the literature, while excluding earlier conventional risk-factor and nomogram studies that were outside the intended scope of this review.

### 2.3 Study selection and data extraction

Two reviewers independently screened titles/abstracts and then full texts using prespecified criteria. Disagreements were resolved by discussion, with third-reviewer adjudication if needed. Ten percent of studies were dual-extracted for calibration; remaining studies were extracted with second-reviewer verification. The data extraction form is provided in Supplementary Material 2.

### 2.4 Risk of bias and applicability appraisal

To assess study quality, we adapted the Quality Assessment of Diagnostic Accuracy Studies-2 (QUADAS-2) tool (18) to align with the objectives of this review. Specifically, we replaced QUADAS-2 items that were not applicable with criteria relevant to AI-based PPH prediction studies (Supplementary Material 3). The modified tool comprised four domains: Participants, Index Test (AI algorithms), Reference Standard (ground truth), and Analysis.

For each domain, we developed four signaling questions tailored to this review. In addition to risk of bias, we assessed applicability for the first three domains (Participants, Index Test, and Reference Standard). The adapted tool was piloted on a subset of ten studies to refine wording and improve calibration. All included studies were then independently assessed by two reviewers using the finalized modified QUADAS-2 tool. Discrepancies were resolved through discussion and consensus.

### 2.5 Data synthesis

We synthesized the extracted data narratively, presenting findings through text, tables, and figures. Synthesis was organized across four domains: (1) Study metadata, design, and population (author, year, publication type, country, research design, number of sites). (2) Postpartum hemorrhage clinical context (clinical applications, predicted outcomes, ground truth, time of prediction). (3) Input data used for AI modeling: data source, number and age of subjects, input data types. (4) AI model characteristics: AI task; model architectures; validation type and technique; and performance metrics used. Outcome categories were assigned according to the clinical construct and modeling target reported in each study. Models predicting the presence or absence of PPH according to each study's operational definition were classified as PPH occurrence. Models predicting severe-threshold outcomes or categorical hemorrhage severity were classified as PPH severity/severe hemorrhage, whereas models predicting blood loss volume in milliliters were classified as quantitative blood loss. Composite endpoints incorporating PPH with transfusion, interventions, or severe maternal morbidity indicators were classified as composite adverse outcomes. A formal meta-analysis was not performed because the included studies were methodologically and clinically heterogeneous with respect to PPH definitions, reference standards, prediction windows, and reported performance measures, and because several studies did not provide the threshold-specific or confusion-matrix data required for robust quantitative synthesis.

## 3 Results

### 3.1 Search results

**Figure 1:**
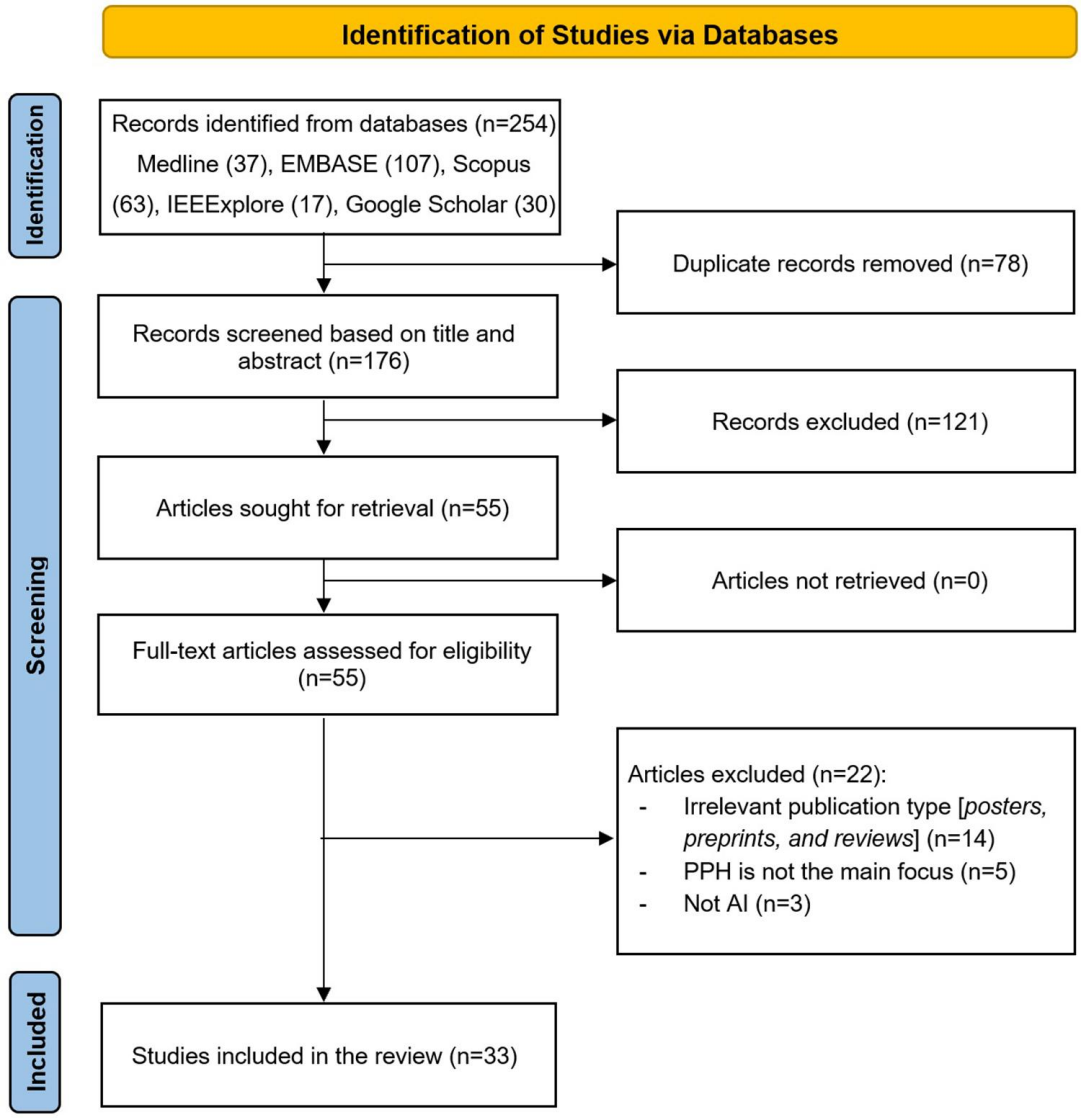
Prisma flow diagram of study selection process.

As shown in Figure 1, a total of 254 records were identified through database searches (MEDLINE 37, Embase 107, Scopus 63, IEEE Xplore 17, Google Scholar 30). After removing 78 duplicates, 176 records remained for title and abstract screening, of which 121 records were excluded. The full texts of 55 articles were retrieved and assessed for eligibility. Of these, 22 articles were excluded for the following reasons: irrelevant publication type (posters, preprints, and reviews; *n* = 14), PPH not the main focus (*n* = 5), and not AI (*n* = 3). Finally, 33 studies were included in the review (19–51).

### 3.2 Characteristics of the included studies

**Figure 2:**
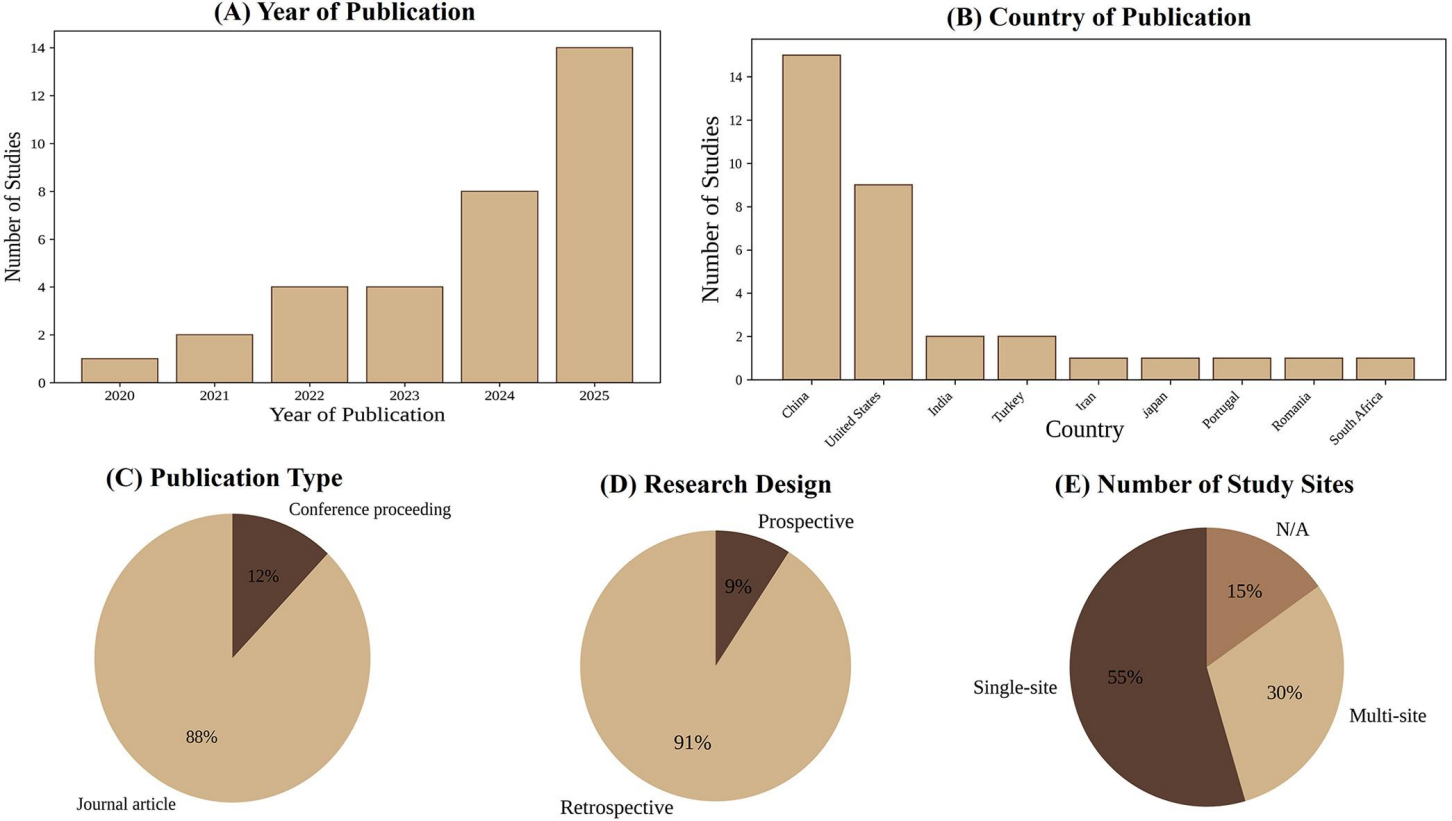
Characteristics of the included studies: **(A)** year of publication; **(B)** country of publication; **(C)** publication type; **(D)** research design; and **(E)** number of study sites.

While studies throughout the years 2020-2025 were included, publications were heavily concentrated in 2023–2025, with 26 of 33 studies (79%) published during this period (2025: 14 studies, 2024: 8 studies, 2023: 4 studies), reflecting rapidly emerging interest and accelerating research activity in AI-based PPH prediction (Figure 2A). Studies originated from nine countries, most commonly China (15/33, 45%) and the United States (9/33, 27%), followed by India and Turkey (each 2/33, 6%) (Figure 2B). The majority were journal articles (29/33, 88%), with conference proceedings accounting for 4 studies (12%). Methodologically, study designs were predominantly retrospective (30/33, 91%), with only three prospective studies (9%). With respect to study setting, single-site studies predominated (18/33, 55%), followed by multi-site studies (10/33, 30%); five studies (15%) did not report the number of sites. Detailed per-study meta-data and research design characteristics are available in Supplementary Material 4.

### 3.3 Postpartum hemorrhage prediction context

Across the included studies (Table 1), most AI models were designed for anticipatory risk stratification before hemorrhage onset, reported in 26 studies (79%). Smaller subsets focused on early warning or severity escalation during labor or the immediate postpartum period (8/33; 24%) and automated case identification/diagnosis using documentation-derived phenotyping (3/33; 9%), with some studies addressing more than one application. Ground truth definitions were most commonly blood-loss–based (e.g., EBL/QBL thresholds or lab proxies) in 19 studies (58%), followed by documentation-derived phenotypes based on diagnoses/codes or chart review (7/33; 21%) and composite clinical outcomes incorporating transfusion, interventions, or severe maternal morbidity features (5/33; 15%). The primary prediction target was PPH occurrence (26/33; 79%), while fewer studies predicted severe PPH/severe hemorrhage (3/33; 9%), composite adverse outcomes (2/33; 6%), quantitative blood loss in milliliters (1/33; 3%), or PPH phenotypes/etiology subtypes (1/33; 3%). Outcomes were predominantly modeled as binary classifications (30/33; 91%), with limited use of categorical (3/33; 9%) or continuous outcomes (1/33; 3%). In terms of prediction timing, nearly half of studies generated predictions intrapartum or peripartum (16/33; 48%), with fewer models operating pre-delivery (8/33; 24%) or at labor admission/triage (5/33; 15%); early postpartum prediction (0–2 hours) and postpartum documentation-based prediction (≥24 hours) were each reported in one study (3%). Detailed per-study postpartum hemorrhage prediction context are available in Supplementary Material 5.

**Table 1:** Postpartum hemorrhage prediction context across included studies.

Feature	Number of Studies (%)	Study IDs
**Clinical Application**		
A) Anticipatory risk stratification (pre-event)	26 (79%)	(19, 20, 22–29, 31, 34–41, 43, 45–48, 50, 51)
B) Early warning/severity escalation (intrapartum or immediate postpartum)	8 (24%)	(30, 32, 33, 42, 44, 48, 49, 51)
C) Automated case identification/diagnosis (documentation-derived phenotyping)	3 (9%)	(21, 28, 49)
**Ground Truth**		
Blood-loss–based (EBL/QBL/quantified blood loss/lab proxy)	19 (58%)	(20, 26, 29, 31–33, 35, 37–40, 42, 44–50)
Documentation-derived phenotype (diagnosis/codes/chart review/labels)	7 (21%)	(21, 22, 24, 30, 34, 41, 43)
Composite clinical outcome (transfusion/interventions/signs/SMM)	5 (15%)	(19, 23, 25, 28, 51)
N/A	2 (6%)	(27, 36)
**Outcome (target)**		
PPH occurrence	26 (79%)	(20, 22–27, 29, 31, 33–40, 43, 44–51)
Composite adverse outcome (PPH + transfusion/interventions/SMM)	2 (6%)	(19, 28)
PPH severity/severe hemorrhage	3 (9%)	(30, 32, 41)
Quantitative blood loss (mL)	1 (3%)	(42)
PPH phenotyping/etiology subtype classification	1 (3%)	(21)
**Outcome Type**		
Binary	30 (91%)	(19–29, 31, 33–41, 43–51)
Categorical	3 (9%)	(21, 30, 32)
Continuous	1 (3%)	(42)
**Time of Prediction**		
Pre-delivery (antepartum/preoperative)	8 (24%)	(19, 25, 31, 37, 45, 46, 50, 51)
Labor admission/triage (pre-delivery baseline)	5 (15%)	(26, 28, 29, 35, 40)
Intrapartum/peripartum (during labor through delivery)	16 (48%)	(19, 20, 23, 24, 33, 34, 37, 38, 40–42, 44, 47–49, 51)
Early postpartum (0–2 hours after delivery)	1 (3%)	(43)
Postpartum (≥24 hours/discharge documentation)	1 (3%)	(21)
N/A	6 (18%)	(22, 27, 30, 32, 36, 39)

### 3.4 Input data used for AI modeling

As summarized in Table 2, all included studies relied on closed, non-public datasets (33/33; 100%), with no studies using open datasets. Across studies reporting sample size (32/33; 97%), cohort sizes were highly variable, with a mean of 29,744 participants (SD 48,853) and a wide range from 147 to 185,413. Participant age was inconsistently reported: 12 studies (36%) provided a mean age (overall mean 31.29 years, SD 1.94) with an age range of 11–58 years, whereas 21 studies (64%) did not report age. With respect to model inputs, most studies incorporated antepartum data (29/33; 88%) and intrapartum data (25/33; 76%), and demographics were included at a similar frequency (29/33; 88%). Postpartum variables were used far less often (5/33; 15%). Notably, these input categories were not mutually exclusive, reflecting frequent integration of multiple data types within individual models. Detailed per-study dataset characteristics are available in Supplementary Material 6.

**Table 2:** Input data used for AI modeling across included studies.

Feature	Number of Studies (%)	Study IDs
**Data Source (Open/closed)**		
Closed	33 (100%)	(19–51)
Open	0 (0%)	-
**Number of Participants**		
Mean number of participants (SD)	29,744 (48,853)	(19–35, 37–51)
Range	147–185,413	
N/A (sample size not reported)	1 (3%)	(36)
**Age of Participants**		
Mean age (SD)	31.29 (1.94)	(23, 25, 26, 29, 37, 39, 41, 44, 45, 46, 50, 51)
Age range	11-58	
N/A	21 (64%)	(19–22, 24, 27, 28, 30–36, 38, 40, 42, 43, 47–49)
**Input Data Used for AI models**		
Antepartum data	29 (88%)	(19–26, 28, 29, 31, 33–35, 37–51)
Intrapartum data	25 (76%)	(19–24, 26, 28, 29, 31, 33, 34, 35, 37–44, 47, 48, 49, 51)
Postpartum data	5 (15%)	(21, 23, 38, 43, 44)
Demographics	29 (88%)	(19–26, 28, 29, 31, 33, 34, 35, 37–51)

### 3.5 AI model characteristics

Most studies relied on classical machine learning approaches (28/33; 85%), while deep learning models were used in 11 studies (33%) and LLM-based approaches in 2 studies (6%), with overlap across categories in some studies. At the algorithm level, logistic regression and random forest were the most frequently implemented methods (each 20/33; 61%), followed by support vector machines (10/33; 30%) and gradient-boosting variants, including XGBoost (9/33; 27%), Gradient Boosting (4/33; 12%), LightGBM (4/33; 12%), and CatBoost (3/33; 9%). Deep neural networks were reported in 3 studies (9%), and ResNet-based architectures appeared in 2 studies (6%), while a diverse set of additional methods were each reported in single studies (12/33; 36%).

In terms of validation, internal validation was most common (25/33; 76%), whereas fewer studies reported combined internal and external validation (4/33; 12%) or external-only validation (3/33; 9%). Hold-out splits were the predominant validation technique (22/33; 67%), with k-fold cross-validation used in 12 studies (36%) and temporal validation in 3 studies (9%). Performance reporting most frequently included AUROC (26/33; 79%), sensitivity (21/33; 64%), and accuracy (20/33; 61%), with F-score and specificity each reported in 12 studies (36%) Table 3. Detailed per-study AI model characteristics are available in Supplementary Material 7.

**Table 3:** AI model characteristics across included studies.

Feature	Number of Studies (%)	Study IDs
**AI Model Family**		
Classical ML	28 (85%)	(19, 20, 23–31, 33–45, 47–49, 51)
Deep learning	11 (33%)	(19, 20, 22, 23, 27, 32, 39, 42, 43, 46, 50)
LLM	2 (6%)	(21, 45)
**Specific AI Architecture**		
Logistic Regression	20 (61%)	(19, 20, 23, 25, 26, 29, 31–38, 40, 44–46, 50, 51)
Random Forest	20 (61%)	(19, 20, 23, 24, 32–42, 44, 47–49, 51)
SVM	10 (30%)	(19, 20, 23, 34, 38, 39, 44, 47, 49, 51)
XGBoost	9 (27%)	(26, 33, 34, 38, 40, 42, 45, 47, 49)
Decision Tree	5 (15%)	(20, 34, 36, 37, 39)
LASSO	5 (15%)	(25, 26, 35, 40, 50)
Gradient Boosting	4 (12%)	(19, 35, 44, 51)
LightGBM	4 (12%)	(33, 34, 38, 47)
Naive Bayes	4 (12%)	(37, 39, 49, 51)
CatBoost	3 (9%)	(30, 38, 47)
Deep Neural Network	3 (9%)	(32, 42, 43)
KNN	3 (9%)	(33, 34, 38)
Neural Network	3 (9%)	(20, 39, 49)
Multilayer Perceptron	2 (6%)	(19, 23)
ResNet	2 (6%)	(46, 50)
Others (each appears in 1 study)	12 (36%)	(20–22, 27, 28, 31, 32, 34, 37, 41, 45, 49)
**Validation Type**		
Internal	25 (76%)	(19–25, 27, 29, 30, 32, 33, 34, 37, 39, 41–49, 51)
Internal + External	4 (12%)	(26, 28, 31, 38)
External	3 (9%)	(35, 40, 50)
N/A	1 (3%)	(36)
**Validation Technique**		
Hold-out split	22 (67%)	(19–23, 25, 26, 28, 31–33, 35, 37, 38, 41, 42, 44, 46, 47, 49–51)
K-fold cross-validation	12 (36%)	(24, 25, 27, 29, 33, 35, 37, 40, 43, 47, 48, 51)
Temporal validation	3 (9%)	(38, 40, 45)
N/A	4 (12%)	(30, 34, 36, 39)
**Performance Metric**		
AUROC	26 (79%)	(19, 20, 23–29, 31, 33–35, 37–41, 43–48, 50, 51)
Sensitivity	21 (64%)	(19, 21–24, 26–31, 33, 34, 37, 39, 43, 44, 46, 47, 49, 50)
Accuracy	20 (61%)	(20–23, 26, 27, 29, 30–32, 34, 36, 37, 39, 44–47, 49, 51)
F-Score	12 (36%)	(19, 21, 26, 27, 29, 30, 39, 41, 43, 47, 49, 51)
Specificity	12 (36%)	(19, 21–25, 29, 33, 34, 37, 46, 50)
PPV	11 (33%)	(19, 21, 23, 25–29, 39, 47, 49)
MCC	4 (12%)	(19, 27, 43, 49)
AUPRC	3 (9%)	(19, 28, 47)
DCA	2 (6%)	(26, 50)
MAE	2 (6%)	(22, 42)
NPV	2 (6%)	(23, 29)
Others (each reported in 1 study)	5 (15%)	(20, 22, 24, 28, 42)

### 3.6 Results of risk of bias appraisal

**Figure 3:**
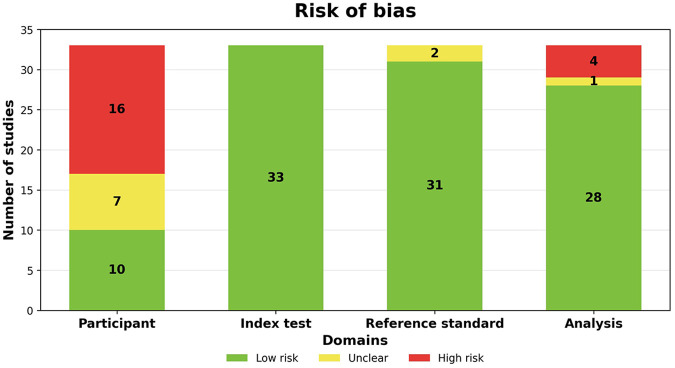
Risk-of-bias assessment across included studies by assessment domain.

**Figure 4:**
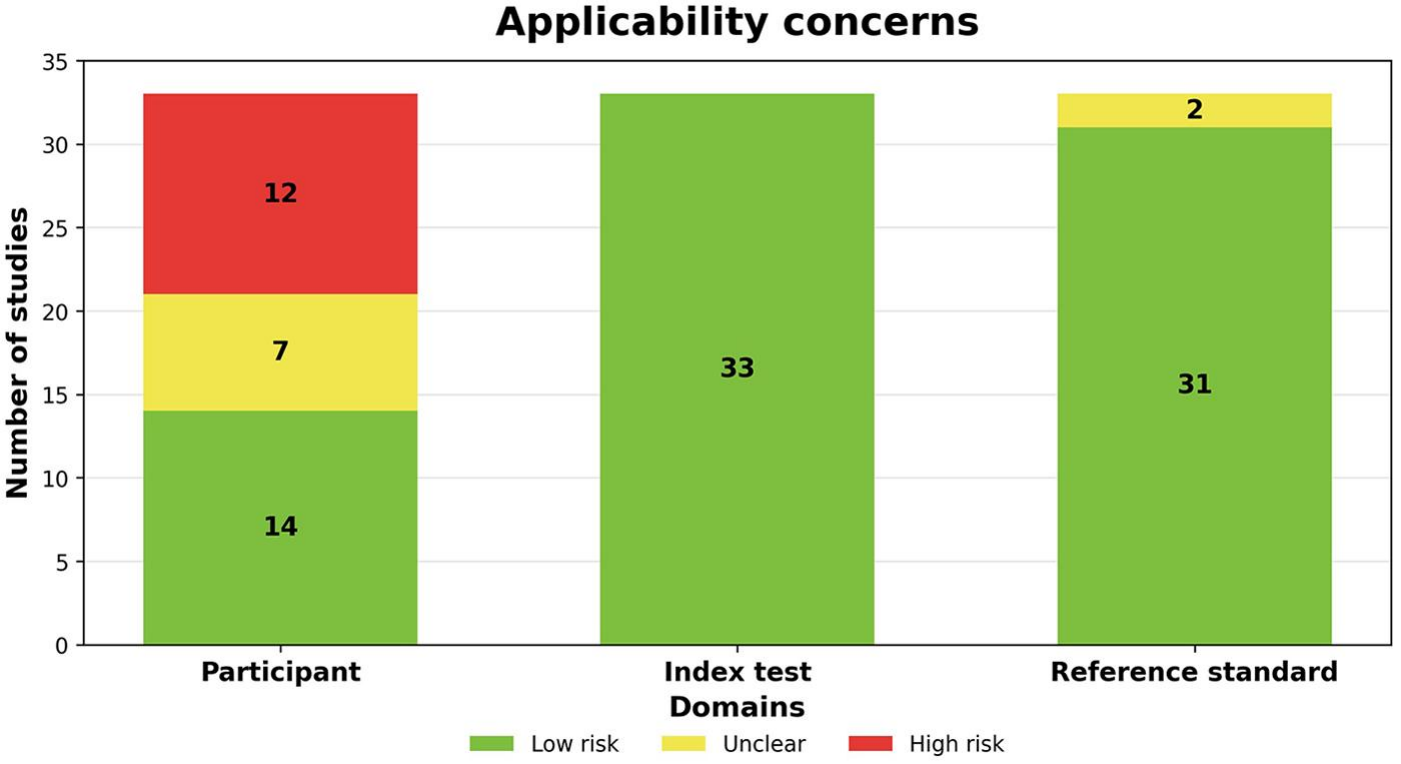
Applicability concerns across included studies by assessment domain.

Risk of bias was assessed using a modified QUADAS-2 tool (Supplementary Material 3) across four domains: participant selection, index test, reference standard, and analysis. A summary of risk of bias judgments is presented in Figure 3, and applicability concerns are summarized in Figure 4. Domain-level ratings for each study are provided in Supplementary Material 8.

Overall, risk of bias was lowest for the index test domain, with all studies rated as low risk (33/33). The reference standard domain was also largely robust, with 31 studies rated low risk and two rated unclear. In contrast, participant selection represented the most common source of bias: 10 studies were rated low risk, seven unclear, and 16 high risk. For the analysis domain, most studies were rated low risk (28/33), while four were judged high risk and one unclear.

Applicability concerns followed a similar pattern. All studies were rated low concern for applicability of the index test (33/33), and most were rated low concern for the reference standard (31/33), with two unclear. Participant applicability showed greater variability, with 14 studies rated low concern, seven unclear, and 12 high concern, indicating that patient populations and sampling approaches were not consistently representative of intended real-world settings.

## 4 Discussion

### 4.1 Main findings

In this systematic review of 33 studies, the evidence base for AI in postpartum hemorrhage prediction was found to be rapidly expanding but still methodologically heterogeneous and largely early-stage in terms of clinical translation. Most included studies were published in recent years and were predominantly retrospective and single-site, suggesting that the field is moving quickly but remains centered on model development rather than deployment-ready evaluation.

Several key patterns emerged across the included studies. First, the dominant prediction setting was anticipatory risk stratification before hemorrhage onset, whereas fewer studies focused on intrapartum, peripartum, or immediate postpartum early warning and severity escalation. This indicates that most current AI models are designed to identify patients at increased risk before overt clinical deterioration, rather than to support dynamic monitoring as hemorrhage risk evolves during labor and delivery. While pre-event models may support preparedness, resource planning, and escalation readiness, they may be less able to capture rapidly developing intrapartum factors that contribute to PPH.

Second, the evidence base was strongly anchored in routinely collected clinical and EHR-derived data from closed datasets. Most studies used antepartum, intrapartum, and demographic variables, whereas postpartum variables and higher-frequency physiologic signals were used less often. This pattern reflects the practical availability of structured obstetric data but also highlights the limited use of more dynamic, multimodal, or real-time data streams that may be important for early warning applications.

Third, classical machine-learning approaches predominated, particularly logistic regression, random forest, support vector machines, and gradient-boosting methods, while deep learning and LLM-based approaches were less frequent. In this review, models were positioned according to their role in PPH prediction rather than algorithmic complexity alone. Regression-based approaches were included only when they were developed, benchmarked, or validated as supervised prediction models, and not when used solely for conventional risk-factor inference or standalone nomogram construction. Accordingly, classical machine-learning models were interpreted as structured-data predictive approaches, whereas deep learning models represented more complex architectures for image, tabular, or augmented-data prediction, and LLM/NLP-based approaches were positioned as emerging methods for extracting or modeling risk-relevant information from clinical text. This distinction supports a clearer interpretation of the evidence, particularly because simpler structured-data models may be more feasible and interpretable in obstetric settings, while more complex models require stronger validation and clearer evidence of incremental clinical value.

Fourth, validation and performance reporting were mainly discrimination-focused and internally oriented. Most studies used internal validation methods such as hold-out splits or cross-validation, while external validation, temporal validation, and decision-impact evaluation were less commonly reported. As a result, many models demonstrate promising predictive performance within development datasets, but their generalizability, calibration, and operational value across different clinical settings remain uncertain.

Finally, our modified QUADAS-2 appraisal showed that the index test and reference standard domains were generally rated as low risk, whereas participant selection was the most frequent source of risk of bias and applicability concern. Taken together, these findings suggest that AI-based PPH prediction has progressed substantially as a research area, but the current evidence is still better characterized as exploratory and development-focused than as ready for broad clinical implementation. Progress toward clinical readiness will require more representative cohorts, harmonized outcome definitions, stronger external and temporal validation, and evaluation strategies aligned with real-world obstetric workflows.

### 4.2 Methodological gaps and translational barriers

#### 4.2.1 Outcome definition, label quality, and clinical construct mismatch

PPH is not a single, uniformly measured endpoint, and included studies operationalized it using diverse reference standards. Blood-loss-based definitions (estimated or quantified) are clinically intuitive but vulnerable to measurement error and documentation variability, particularly when estimated blood loss is used instead of quantified measurement. In contrast, diagnosis codes and chart-derived phenotypes are influenced by institutional documentation and coding practices and may under- or over-capture true physiologic hemorrhage. Composite outcomes that incorporate transfusion or interventions can be clinically meaningful for escalation, but may be partly determined by practice patterns, staffing, and local thresholds rather than bleeding severity alone.

These label choices influence both apparent model performance and clinical meaning. A model optimized to predict coded PPH may learn documentation and billing patterns rather than hemorrhage physiology. Similarly, a model predicting transfusion or intervention-based composites may transport poorly to institutions with different transfusion thresholds, uterotonic protocols, or availability of interventional radiology. A key translational step is therefore explicit alignment between the predicted outcome and the intended clinical action. Where feasible, future studies should report multiple aligned endpoints (e.g., PPH occurrence, severe PPH, transfusion, and escalation) rather than a single heterogeneous label, and should clearly specify measurement methods and timing relative to delivery.

#### 4.2.2 Timing, actionability, and treatment confounding

Prediction timing is central to clinical utility. Pre-delivery and triage models can inform early preparedness but may resemble refined versions of existing risk checklists and may miss events driven by intrapartum processes (e.g., uterine atony evolving during labor, operative events, or complications that develop after admission). Intrapartum and immediate postpartum prediction can leverage higher-frequency signals (vital signs, labor progress, laboratory trends, and emergent complications), potentially enabling earlier recognition of evolving hemorrhage. However, these settings are also most vulnerable to treatment confounding and temporal leakage, because interventions (uterotonics, fluids, operative delivery, manual exploration, transfusion) can both respond to early signs of hemorrhage and shape the recorded predictors and outcomes.

To preserve validity, studies should define explicit prediction timestamps (or a sequence of timestamps), restrict predictors to those available at the time of prediction, and account for time-dependent interventions. Landmarking approaches, dynamic models that update risk with time-stamped features, and separate models aligned to specific decision points (e.g., triage, active labor, immediately post-delivery) can better map predictions to actionable workflows while reducing leakage risk. Clear reporting of what information is “available” at prediction time is particularly important when postpartum variables are included, because these may not be clinically available, or may already reflect the evolving hemorrhage process, depending on the intended use case.

#### 4.2.3 Validation, calibration, and clinically meaningful evaluation

Validation and evaluation practices remain major barriers to clinical transportability. Most studies relied on internal validation, commonly using random hold-out splits or cross-validation. While these approaches are appropriate for initial development, they can misrepresent deployment conditions when data are clustered within hospitals or units, when clinical pathways change over time, or when individuals have multiple encounters. External validation and temporal validation were comparatively uncommon, despite being essential to quantify dataset shift and to support adoption beyond the development setting.

Performance reporting frequently emphasized AUROC and accuracy. In low-prevalence outcomes and class-imbalanced settings, these metrics can obscure the operational consequences of false positives (e.g., unnecessary activation, increased resource use, and alert fatigue). Additionally, several studies have either not provided confidence intervals for AUROC, employed different validation methods, or presented models without a predetermined selection strategy, which constrains the feasibility of meta-analysis. Clinically, models must be evaluated at specific operating points, reporting sensitivity, positive predictive value, and alert rates (e.g., per 100 deliveries), alongside calibration (whether predicted risks match observed probabilities). Utility-oriented evaluations such as decision-curve analysis, and measures that help quantify clinical workload and safety trade-offs, remain underused. These gaps contribute to the “last mile” barrier where promising discrimination does not translate into usable decision support.

#### 4.2.4 Generalizability, equity, and reproducibility

Participant selection was the most frequent source of risk of bias and applicability concern in our appraisal, consistent with a literature dominated by retrospective and single-center designs. Selective inclusion criteria, enrichment for high-risk cohorts, and incomplete capture of transfers or out-of-hospital births can introduce spectrum bias and inflate performance. Even when models are internally validated, differences in case mix, documentation practices, and care pathways across institutions can lead to rapid degradation in real-world settings.

An additional equity concern relates to the geographic concentration of the evidence base. In our review, 45% of included studies originated from China and 27% from the United States, indicating that current AI development for PPH prediction is largely driven by higher-resource settings. This concentration may limit applicability to low- and middle-income countries (LMICs), where the burden of postpartum hemorrhage is greatest and where clinical workflows, case mix, resource availability, blood product access, documentation practices, and digital infrastructure may differ substantially from the settings in which these models were developed. As a result, models trained and validated primarily in China- and U.S.-based datasets may not generalize well to LMIC contexts without local adaptation and validation. Future work should therefore prioritize geographically diverse cohorts, multicenter collaborations that include LMIC settings, and equity-oriented evaluation to ensure that AI tools for PPH prediction do not widen existing global maternal health disparities.

Finally, the exclusive reliance on closed datasets limits reproducibility, independent benchmarking, and transparent error analysis. Maternal health data governance constraints are real, but they strengthen the case for shared benchmarking strategies such as multicenter consortia, standardized reporting templates, and federated evaluation frameworks that allow independent testing without centralizing patient-level data. Establishing common data definitions and evaluation protocols would enable more meaningful comparisons across models and accelerate progress toward clinically trustworthy systems.

### 4.3 Future directions for AI in postpartum hemorrhage

**Figure 5:**
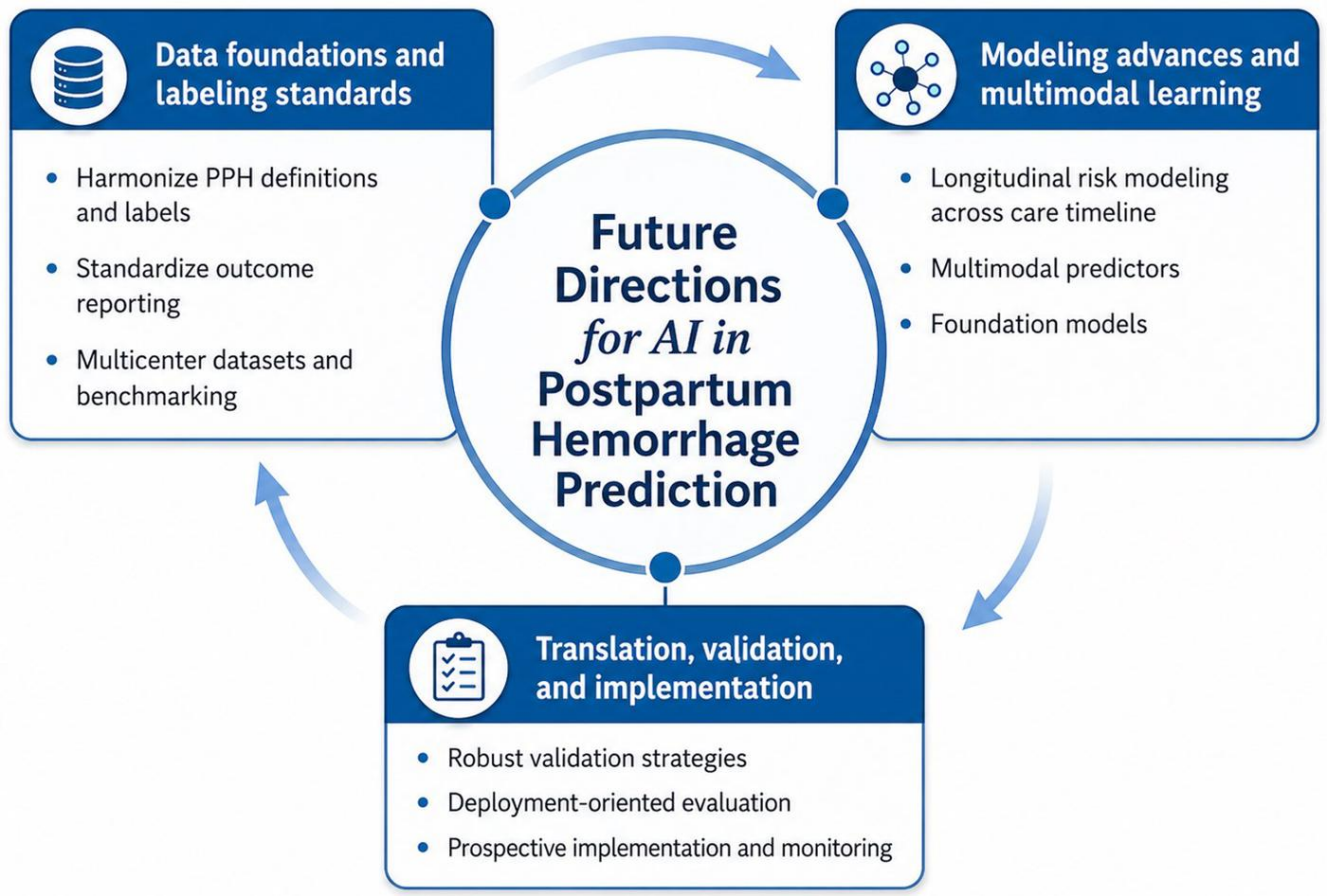
Future directions for AI in postpartum hemorrhage.

Future work should shift from proof-of-concept modeling toward deployment-ready evidence, with priorities spanning data foundations, modeling strategy, evaluation, and implementation science (Figure 5).

First, the field would benefit from harmonized label definitions and reporting standards that explicitly distinguish between physiologic hemorrhage (blood loss and laboratory proxies), treated hemorrhage (transfusion and interventions), and documentation-derived phenotypes. A pragmatic approach is to support multiple aligned targets (e.g., PPH occurrence, severe PPH, transfusion, and escalation) and to report timing relative to delivery. This would facilitate matching models to concrete escalation pathways and would help avoid conflating physiologic bleeding with practice-pattern outcomes.

Second, methodological development should emphasize longitudinal and multimodal models that capture dynamic labor processes. This includes time-stamped vital signs and laboratory trends, medication and intervention timelines, delivery mode and operative events, and, when available, bedside device signals, waveform-derived features, or imaging. Models should explicitly handle missingness and irregular sampling, which are common in obstetric data and can be informative if managed transparently. When deep learning is used, comparative evaluation against simpler baselines should be paired with interpretability approaches that support clinician trust and error analysis.

Third, translation requires stronger validation and clinically aligned evaluation. Models intended for clinical use should undergo temporal validation and external validation across settings that differ in patient mix and practice patterns. Reporting should include calibration, threshold-based operating points, and utility-oriented measures such as net benefit, along with detailed error analyses that identify failure modes by delivery mode, hemorrhage etiology, comorbidity profiles, and care setting. Where possible, studies should compare performance against existing clinical tools or standard-of-care risk stratification approaches to clarify incremental value.

Fourth, implementation studies are essential. Prospective silent trials can quantify drift and calibration without affecting care, and can inform threshold selection and user-interface design. Subsequent stepped-wedge or cluster-randomized evaluations can measure clinical outcomes, workflow impact, equity, and unintended consequences (including alert fatigue and overtreatment). Post-deployment monitoring should be pre-specified, with governance for recalibration, drift detection, and model updating over time.

Finally, emerging foundation-model and LLM-based approaches may contribute by extracting structured risk signals from free-text notes and improving data efficiency through transfer learning. However, these approaches must be evaluated with the same rigor as other predictive systems, including stability, calibration, and real-world failure modes, and should not be positioned as clinically ready without prospective evidence.

### 4.4 Review limitations

This systematic review has limitations. We restricted inclusion to English-language publications indexed in selected databases through December 2025; relevant work in other languages, preprints, or grey literature may have been missed. Meta-analysis was not feasible because outcomes, reference standards, prediction timing, and performance reporting were highly heterogeneous, and key elements needed for quantitative pooling (e.g., consistent thresholds and complete confusion-matrix reporting) were often unavailable. In addition, our risk-of-bias assessment used a modified QUADAS-2 tool tailored to AI-based PPH prediction. While this improves domain relevance, judgments remain dependent on reporting completeness, and some studies may have employed stronger practices than could be inferred from published descriptions alone.

## 5 Conclusion

AI-based prediction for postpartum hemorrhage is a rapidly expanding field, with most studies focusing on pre-event risk stratification using retrospective, closed datasets and primarily internal validation. Although many models report favorable discrimination, heterogeneity in outcome definitions, limited postpartum feature use, and frequent concerns related to participant selection constrain generalizability and clinical readiness. Progress toward real-world impact will require harmonized PPH labeling, multicenter datasets, stronger external and temporal validation, and deployment-oriented evaluation emphasizing calibration and clinical utility. Emerging multimodal and foundation-model approaches offer strong potential to advance PPH prediction, and well-designed prospective implementation studies will be valuable to confirm their safety, effectiveness, and long-term sustainability in real-world practice.

## Data Availability

The original contributions presented in the study are included in the article/Supplementary Material, further inquiries can be directed to the corresponding author.
