# Supplementary material for: Artificial intelligence for postpartum hemorrhage: a systematic review
Source: Front Glob Womens Health. 2026 Jun 12;7:1806220. doi: 10.3389/fgwh.2026.1806220 (PMC13303495; doi:10.3389/fgwh.2026.1806220)
Supplement: Supplementary file 1 [file Supplementaryfile1.pdf]

Supplementary Material 1: Search strategy

Database(s): **Ovid MEDLINE(R) ALL** 1946 to December 19, 2025
Search Strategy:

#	Searches	Results
1	exp Artificial Intelligence/	264069
2	"artificial intelligence".tw.	78722
3	exp Machine Learning/	111311
4	"machine learning".tw.	153929
5	exp Deep Learning/	37280
6	"deep learning".tw.	87364
7	exp Postpartum Hemorrhage/	9383
8	"postpartum hemorrhag*".tw.	6958
9	"postpartum haemorrhag*".tw.	2583
10	"post partum hemorrhag*".tw.	661
11	"post partum haemorrhag*".tw.	704
12	"post-partum hemorrhag*".tw.	661
13	"post-partum haemorrhag*".tw.	704
14	"postpartum bleed*".tw.	530
15	"postpartum blood loss".tw.	291
16	"obstetric hemorrhag*".tw.	891
17	"obstetric haemorrhag*".tw.	467
18	1 or 2 or 3 or 4 or 5 or 6	413753
19	7 or 8 or 9 or 10 or 11 or 12 or 13 or 14 or 15 or 16 or 17	15429
20	18 and 19	52

21	limit 20 to english language	52
22	limit 21 to humans	38
23	limit 22 to yr="2015 - 2025"	37

Database(s): **Embase** 1974 to 2025 Week 51
Search Strategy:

#	Searches	Results
1	exp Artificial Intelligence/	174717
2	"artificial intelligence".tw.	94445
3	exp Machine Learning/	678257
4	"machine learning".tw.	180941
5	exp Deep Learning/	101184
6	"deep learning".tw.	102131
7	exp Postpartum Hemorrhage/	23548
8	"postpartum hemorrhag*".tw.	10562
9	"postpartum haemorrhag*".tw.	4632
10	"post partum hemorrhag*".tw.	1398
11	"post partum haemorrhag*".tw.	1305
12	"post-partum hemorrhag*".tw.	1398
13	"post-partum haemorrhag*".tw.	1305
14	"postpartum bleed*".tw.	833
15	"postpartum blood loss".tw.	450
16	"obstetric hemorrhag*".tw.	1385
17	"obstetric haemorrhag*".tw.	1131

18	1 or 2 or 3 or 4 or 5 or 6	785242
19	7 or 8 or 9 or 10 or 11 or 12 or 13 or 14 or 15 or 16 or 17	27481
20	18 and 19	217
21	limit 20 to english language	216
22	limit 21 to humans	212
23	limit 22 to yr="2015 - 2025"	209
24	limit 23 to "remove medline records"	107

Database	Query	Results
**Scopus**	(TITLE-ABS ("artificial intelligence") OR TITLE-ABS ("machine learning") OR TITLE-ABS ("deep learning")) AND (TITLE-ABS ("postpartum hemorrhag*") OR TITLE-ABS ("postpartum haemorrhag*") OR TITLE-ABS ("post partum hemorrhag*") OR TITLE-ABS ("post partum haemorrhag*") OR TITLE-ABS ("post-partum hemorrhag*") OR TITLE-ABS ("post-partum haemorrhag*") OR TITLE-ABS ("postpartum bleed*") OR TITLE-ABS ("postpartum blood loss") OR TITLE-ABS ("obstetric hemorrhag*") OR TITLE-ABS ("obstetric haemorrhag*"))	63
**IEEE Xplore**	("All Metadata":"artificial intelligence" OR "All Metadata":"machine learning" OR "All Metadata":"deep learning")<br>AND<br>(<br>"All Metadata":"postpartum hemorrhag*" OR<br>"All Metadata":"postpartum haemorrhag*" OR<br>"All Metadata":"post partum hemorrhag*" OR<br>"All Metadata":"post partum haemorrhag*" OR<br>"All Metadata":"post-partum hemorrhag*" OR	17

	"All Metadata":"post-partum haemorrhag*" OR<br>"All Metadata":"postpartum bleed*" OR<br>"All Metadata":"postpartum blood loss" OR<br>"All Metadata":"obstetric hemorrhag*" OR<br>"All Metadata":"obstetric haemorrhag*"<br>)	
**Google Scholar (Labs)**	("artificial intelligence" OR "machine learning" OR "deep learning")<br>AND ("postpartum hemorrhage" OR "postpartum haemorrhage" OR "post partum hemorrhage" OR "post partum haemorrhage" OR "post-partum hemorrhage" OR "post-partum haemorrhage" OR "postpartum bleeding" OR "postpartum blood loss" OR "obstetric hemorrhage" OR "obstetric haemorrhage" OR PPH)	30

Supplementary Material 2: Data Extraction Form

1. Study Metadata, Study Design, Population Characteristics

**Field name**	**Description**	**Possible values**
ID	Unique study identifier in your review	Numeric or alphanumeric
Author	First author surname	Free text
Year	Publication year	YYYY
Type of publication	Publication type	Journal article; Conference proceeding/article
Country	Country of study setting (prefer study cohort country; if unclear, use first author affiliation country)	Free text; if multi-country, list all
Research design (Retrospective, Prospective)	Timeline of data collection	Retrospective; Prospective; Mixed/Hybrid; Not reported
Number of sites (clinics/hospitals)	Distinct sites contributing data	Single-center; Multi-center; Not reported (+ count if given)
Number of subjects	Total participants/deliveries included in modeling	Integer; specify exclusions if reported
Number of cases		
Number of controls		
Mean Age	Mean maternal age (or median if mean not available)	Mean±SD or median (IQR); Not reported

2. PPH Phenotype & Staging/ Clinical Context

Field name	Description	Possible values
PPH definition	The exact operational definition used for PPH labeling	(e.g., "EBL ≥1000 mL within 24h" / "QBL ≥1000 mL" / "≥500 mL vaginal, ≥1000 mL CS" / "PPH ICD codes")
Reference standard	what source establishes the ground truth (e.g., QBL/EBL thresholds, transfusion criteria, ICD codes, chart review, NLP phenotype).	▪ Blood-loss–based (EBL/QBL/quantified blood loss/lab proxy)<br>▪ Composite clinical outcome (transfusion/interventions/signs/SMM)<br>▪ Documentation-derived phenotype (diagnosis/codes/chart review/labels)<br><br>**EBL** = **Estimated Blood Loss**<br>• A clinician's estimate of how much blood was lost (often visual estimation, sometimes with rough adjustments).<br>**QBL** = **Quantitative Blood Loss**<br>• A more **measured** approach to blood loss, usually by **collecting blood** and **weighing** soaked materials (gravimetric/volumetric methods) to calculate mL lost.
Clinical Application 1	Primary clinical use-case	A) Anticipatory risk stratification (pre-event) Clinical purpose: identify who is at high risk for PPH before it happens so teams can plan (blood products, uterotonics, senior staff, OR readiness), often at admission or pre-cesarean, including high-risk subcohorts (previa/PAS).<br><br>B) Early warning / severity escalation (intrapartum or immediate postpartum) Clinical purpose: detect deterioration or escalating hemorrhage severity around delivery to trigger rapid response (massive transfusion protocol, escalation, interventions). This includes explicit early warning and severity prediction/estimation.<br><br>C) Automated case identification / diagnosis (documentation-derived phenotyping) Clinical purpose: identify or label PPH cases from EHR data (often text/NLP/LLM) for surveillance, audit/quality improvement,

		research cohort building, or automated documentation support (not forecasting risk).
Clinical Application 2	Secondary use-case (if any)	Same categories as above; N/A
Outcome (target)	What the model predicts (must be PPH-related)	▪ PPH occurrence (binary) *(includes proxy definitions such as Hb drop; includes different PPH definitions like EBL/QBL/cPPH)*<br>▪ Composite adverse outcome (PPH + transfusion/interventions/SMM)<br>▪ PPH severity / severe hemorrhage (categorical or severe-threshold binary)<br>▪ Quantitative blood loss (continuous; mL)<br>▪ PPH phenotyping / etiology subtype classification
Outcome Type (binary/multiclass)	Label structure	Binary; Multiclass; Continuous (if present, specify)
Time of Prediction	When prediction is intended to be made	• **Pre-delivery (antepartum / preoperative)**<br>Anything **before labor/delivery**, including **prenatal notes**, **prenatal MRI**, and **pre-cesarean / preoperative assessment** (even "within 1 week").<br>• **Labor admission / triage (pre-delivery baseline)**<br>Explicitly **at admission to labor ward / labor & delivery** (T0, at admission, before delivery).<br>• **Intrapartum / peripartum (during labor through delivery)**<br>Anything **during labor** and/or **around the time of delivery**, including "at delivery," "perioperative variables during cesarean," "second stage," "C-section decision," and "~2h before delivery" uterine contraction curve.<br>• **Early postpartum (0–2 hours after delivery)**<br>Explicit early postpartum window (≤2h).

		• **Postpartum (≥24 hours / discharge documentation)** Later postpartum, including discharge summaries and ≥24h post-delivery.<br>• **Not reported** Use N/A.

3. Dataset Characteristics

**Field name**	**Description**	**Possible values**
Data Source (Open/closed)	Whether data are publicly accessible	Open; Closed; Mixed; Not reported
antepartum data	Inputs include antepartum features?	Yes/No; if Yes: examples (prenatal labs, comorbidities, imaging, antenatal complications)
Intrapartum data	Inputs include labor/delivery features?	Yes/No; if Yes: examples (mode of delivery, oxytocin, vitals, anesthesia, EBL updates)
Demographics	Inputs include demographics?	Yes/No; if Yes: list (age, parity, BMI, ethnicity, etc.)
Postpartum data	Inputs include postpartum features? (generally avoid leakage; note if used)	Yes/No; if Yes: specify timing to assess leakage risk

4. AI Model Characteristics

**Field name**	**Description**	**Possible values**
Model Family (Category)	High-level model type	Classical ML (LR/regularized LR, RF, XGBoost, SVM, etc.); Deep learning (MLP/CNN/RNN); NLP/LLM; Radiomics/Imaging ML; Generative AI (GAI); Large

		Language Models (LLMs); Other (specify).<br>List model families separated by comma.
Specific architecture	Concrete algorithm(s) used	Free text (e.g., XGBoost, Random Forest, MLP, CNN, Transformer/Flan-T5, radiomics+GBM, etc.)
Validation Type	Where validation data comes from relative to training	Internal; External; Internal+External
Validation technique (Categorized)	Evaluation design	▪ Hold-out split (Repeated hold-out, random train/test or train/val split)<br>▪ K-fold cross-validation<br>▪ Temporal validation<br>▪ N/A
Performance metrics	Metrics reported (normalize naming)	AUROC; AUPRC; Accuracy; F1; Sensitivity/Recall; Specificity; PPV; NPV; Calibration (Brier, slope/intercept); Decision curve/Net benefit; MCC; Youden; Other (specify)
Confusion Matrix	Whether TP/FP/TN/FN (or confusion matrix) is reported	Yes; No
Performance Results for all metrics	Extract numeric values for each reported metric	Free text or structured (e.g., AUROC=0.xx [95% CI], etc.); include CI if available

Supplementary Material 3: The modified version of QUADAS-2

1. Participants (Patient Selection)

Signaling Questions	Explanation
**1.1 Was a consecutive or random sample of patients enrolled?**	- **Yes**: If a consecutive or random sample of eligible participants (pregnant women) was enrolled.<br>- **No**: If patients were selected based on convenience or unclear selection criteria.<br>- **Unclear**: If the study did not report participant selection details.
**1.2 Did the study avoid inappropriate exclusions?**	- **Yes**: If inclusion/exclusion criteria were appropriate, ensuring a representative sample of postpartum hemorrhage patients.<br>- **No**: If participants were excluded in a way that could bias model performance (e.g., excluding high-risk groups).<br>- **Unclear**: If no details were provided.
**1.3 Was the sample size sufficient?**	- **Yes**: If at least 100 subjects were included, or if studies with multiple samples per subject had at least 100 samples.<br>- **No**: If fewer than 100 participants or samples were included.<br>- **Unclear**: If the sample size was not clearly reported.
**1.4 Was there a balance in the number of patients across relevant subgroups?**	- **Yes**: If no subgroup comprised more than 75% of the sample.<br>- **No**: If any subgroup dominated the sample.<br>- **Unclear**: If subgroup proportions were not reported.
**Risk of Bias Assessment: Could the selection of participants introduce bias?**<br>• **Low risk of bias**: If all signaling questions are **"Yes."**<br>• **High risk of bias**: If any answer is **"No."**<br>• **Unclear risk of bias**: If key information is missing.	
**Applicability Concerns: Do the participants match the review question?**<br>• **Low concern**: If the study population aligns with the target population of the review.	

- **High concern**: If the study includes a non-representative population (e.g., excluding high-risk pregnancies).
- **Unclear concern**: If insufficient details are provided.

2. Index Test (AI Models)

Signaling Questions	Explanation
**2.1 Were the AI models described in detail?**	- **Yes**: If the study provided technical details (e.g., model type, architecture, hyperparameters, features used).<br>- **No**: If only the model name was reported, or key details were missing.<br>- **Unclear**: If insufficient information was provided.
**2.2 Were all features (predictors) clearly identified?**	- **Yes**: If all features (clinical, biochemical, wearable data, etc.) were explicitly listed.<br>- **No**: If feature selection was unclear or incomplete.<br>- **Unclear**: If no details were provided.
**2.3 Were features assessed in the same way for all participants?**	- **Yes**: If all participants underwent standardized data collection.<br>- **No**: If different methods/devices were used without standardization (e.g., multiple types of glucose monitors with inconsistent calibration).<br>- **Unclear**: If assessment methods were not reported.
**2.4 Were features collected without knowledge of the outcome?**	- **Yes**: If AI predictors were selected independently of outcome data.<br>- **No**: If outcome data influenced feature selection.<br>- **Unclear**: If no details were provided.

Risk of Bias Assessment: Could the index test introduce bias?

- **Low risk of bias**: If all signaling questions are **"Yes."**
- **High risk of bias**: If any answer is **"No."**
- **Unclear risk of bias**: If key information is missing.

Applicability Concerns: Does the index test match the review question?

- **Low concern**: If the AI model aligns with postpartum hemorrhage prediction/diagnosis/treatment monitoring.
- **High concern**: If the model is not directly relevant to the review question.
- **Unclear concern**: If model applicability is not clear.

3. Reference Standard (Ground Truth)

Signaling Questions	Explanation
**3.1 Was the reference standard likely to correctly classify the outcome (postpartum Hemorrhage)**	**- Yes:** If PPH was defined using a **clear, prespecified, clinically accepted definition** with an explicit **time window** (e.g., within 24 h postpartum) and **objective criteria**, such as: (i) **quantified blood loss (QBL)** or documented blood-loss thresholds (e.g., ≥500 mL vaginal / ≥1000 mL cesarean, or ≥1000 mL regardless of mode) **and/or** bleeding with **signs/symptoms of hypovolemia**; (ii) a **validated composite** reflecting clinically significant hemorrhage (e.g., transfusion and/or hemorrhage-specific interventions) with explicit component definitions; (iii) **documentation-derived phenotype** supported by **chart review/adjudication** or validated coding/labeling rules (e.g., ICD codes plus confirmation rules).<br>**- No:** If the "PPH" label relied on **non-validated or vague criteria** (e.g., clinician impression only, undocumented thresholds, unclear timing, unverified administrative codes alone without any validation/check), or if the reference standard was clearly prone to systematic misclassification.<br>**- Unclear:** If the reference standard was **not described sufficiently** (no threshold, no timing, no rule for labeling, no adjudication/validation described).
**3.2 Was the outcome determined in a consistent manner for all participants?**	- **Yes**: If the same diagnostic criteria were applied to all participants.<br>- **No**: If different criteria were applied inconsistently.<br>- **Unclear**: If criteria were not reported.

**3.3 Was the outcome determined without knowledge of predictor data?**	- **Yes**: If outcome classification was blinded to AI model predictions.<br>- **No**: If assessors had access to AI-generated predictions when classifying outcomes.<br>- **Unclear**: If blinding was not reported.
**3.4 Was there a time interval between predictor assessment and outcome determination that could introduce bias?**	**- Yes**: If predictor variables were collected before or up to the intended prediction time point (e.g., antepartum, at labor admission, intrapartum), and the PPH outcome was ascertained within a clinically appropriate postpartum window consistent with the study definition (commonly during delivery through the first 24 hours postpartum, or clearly specified early postpartum window such as 0–2 hours). The timeline is clearly stated and avoids using information that occurs after hemorrhage onset to define predictors.<br>**- No:** If predictors included post-event information (e.g., interventions, transfusion, postpartum labs/vitals after bleeding began) while still being treated as “predictors,” or if the outcome window was misaligned/ill-defined (e.g., outcome determined far beyond the stated window without justification), increasing risk of reverse causality or misclassification.<br>**- Unclear**: If the paper does not clearly report when predictors were measured relative to delivery/PPH onset and/or does not specify the outcome ascertainment window.
**Risk of Bias Assessment: Could the reference standard introduce bias?**<br>• **Low risk of bias**: If all signaling questions are **"Yes."**<br>• **High risk of bias**: If any answer is **"No."**<br>• **Unclear risk of bias**: If key information is missing.	
**Applicability Concerns: Does the reference standard match the review question?**<br>• **Low concern**: If the reference standard is appropriate for postpartum hemorrhage.<br>• **High concern**: If the reference standard is inappropriate (e.g., inconsistent diagnostic thresholds).<br>• **Unclear concern**: If reference standard details are unclear.	

4. Analysis

Signaling Questions	Explanation
**4.1 Were all participants included in the analysis?**	- **Yes**: If no participants were inappropriately excluded.<br>- **No**: If participants were excluded without justification.<br>- **Unclear**: If inclusion/exclusion criteria for analysis were unclear.
**4.2 Was data preprocessing carried out appropriately?**	- **Yes**: If missing data was handled appropriately (e.g., imputation).<br>- **No**: If data preprocessing methods were flawed or not reported.<br>- **Unclear**: If no details were provided.
**4.3 Was the breakdown of training, validation, and test sets appropriate?**	- **Yes**: If data was split using best practices (e.g., 70-80% training, 10-15% validation, 10-20% test).<br>- **No**: If an inappropriate split was used.<br>- **Unclear**: If data splitting details were missing.
**4.4 Was the performance of the model evaluated appropriately?**	**- Yes:** If the confusion matrix was presented,<br>Or more than one measure was used and the selected measures were appropriate.<br>**- No:** If the confusion matrix was not presented, and only one measure was reported,<br>Or the selected measures were not appropriate.<br>**- Unclear:** If no information was provided on the performance measures

Risk of Bias Assessment: Could the analysis introduce bias?

- **Low risk of bias**: If all signaling questions are **"Yes."**
- **High risk of bias**: If any answer is **"No."**
- **Unclear risk of bias**: If key information is missing.

Supplementary Material 4: Per-study Metadata and Design Characteristics

First Author	Year	Type of publication	Country	Research design	Number of sites (clinics/hospitals)

Ahmadzia (2024) [19]	2024	Journal article	United States	Retrospective	Multi
Akazawa (2021) [20]	2021	Journal Article	Japan	Retrospective	Single
Alsentzer (2023) [21]	2023	Journal Article	United States	Retrospective	Multi
Ambeth Kumar (2022) [22]	2022	Journal Article	India	Retrospective	N/A
Dogru (2025) [23]	2025	Journal Article	Turkey	Retrospective	Single
Holcroft (2024) [24]	2024	Journal Article	South Africa	Retrospective	Multi
Hong (2025) [25]	2025	Journal Article	China	Retrospective	Single
Kovacheva (2025) [26]	2025	Journal Article	United States	Retrospective	Multi
Krishnamoorthy (2022) [27]	2022	Journal Article	China	Retrospective	N/A
Lengerich (2024) [28]	2024	Journal Article	United States	Retrospective	Multi
Lérias-Cambeiro (2025) [29]	2025	Journal Article	Portugal	Retrospective	Single
Li (2024) [30]	2024	Conference Proceedings	China	Retrospective	Single
Li (2025a) [31]	2025a	Journal Article	China	Retrospective	Multi
Li (2025b) [32]	2025b	Journal Article	China	Retrospective	Single
Liu (2022) [33]	2022	Journal Article	China	Retrospective	Single
Mehrnoush (2023) [34]	2023	Journal Article	Iran	Retrospective	Single
Meyer (2024) [35]	2024	Journal Article	United States	Retrospective	Single
Raman (2025) [36]	2025	Conference Proceedings	India	Retrospective	N/A
Shah (2023) [37]	2023	Journal Article	United States	Prospective	N/A
Song (2025) [38]	2025	Journal Article	China	Retrospective	Multi
Susanu (2024) [39]	2024	Journal Article	Romania	Prospective	Multi
Venkatesh (2020) [40]	2020	Journal Article	United States	Retrospective	Multi
Wang (2024a) [41]	2024a	Journal Article	China	Retrospective	Single

Wang (2024b) [42]	2024b	Journal Article	China	Retrospective	Single
Wang (2025) [43]	2025	Journal Article	China	Retrospective	Single
Westcott (2022) [44]	2022	Journal Article	United States	Retrospective	Single
Woo (2025) [45]	2025	Journal Article	United States	Retrospective	N/A
Xiao (2025) [46]	2025	Conference Proceedings	China	Retrospective	Single
Yao (2025) [47]	2025	Journal Article	China	Prospective	Single
Yuan (2023) [48]	2023	Conference Proceedings	China	Retrospective	Single
Zhang (2021) [49]	2021	Journal Article	China	Retrospective	Single
Zhang (2025) [50]	2025	Journal Article	China	Retrospective	Multi
Zorlu (2025) [51]	2025	Journal Article	Turkey	Retrospective	Single

Supplementary Material 5: Per-study Postpartum Hemorrhage Prediction Context

Study	PPH Definition	Clinical Application	Ground Truth	Outcome	Outcome Type	Time of Prediction
Ahmadzia (2024) [19]	EBL ≥1000 mL (≥1 L) within 24h of delivery (ACOG reVITALize; documented EBL during/after delivery)	A) Anticipatory risk stratification (pre-event)	Composite clinical outcome (transfusion/interventions/signs/SMM)	Composite adverse outcome (PPH + transfusion/interventions/SMM)	Binary	Pre-delivery (antepartum / preoperative); Intrapartum / peripartum (during labor through delivery)
Akazawa (2021) [20]	Blood loss ≥1000 mL within 24 h after vaginal delivery	A) Anticipatory risk stratification (pre-event)	Blood-loss–based (EBL/QBL /quantified blood loss/lab proxy)	PPH occurrence	Binary	Intrapartum / peripartum (during labor through delivery)
Alsentzer (2023) [21]	EBL >500 mL (vaginal) or >1000 mL (cesarean); primary PPH; phenotype based on LLM-extracted EBL + delivery mode	C) Automated case identification / diagnosis (documentation-derived phenotyping)	Documentation-derived phenotype (diagnosis/codes/chart review/labels)	PPH phenotyping / etiology subtype classification	Binary; Categorical	Postpartum (≥24 hours / discharge documentation)
Ambeth Kumar (2022) [22]	Blood loss >500 mL (vaginal) or >1000 mL (cesarean section) (stated); labeling method not fully described	A) Anticipatory risk stratification (pre-event)	Documentation-derived phenotype (diagnosis/codes/chart review/labels)	PPH occurrence	Binary	N/A
Dogru (2025) [23]	ACOG: >1000 mL blood loss within 24h after birth OR blood loss with signs/symptoms of hypovolemia	A) Anticipatory risk stratification (pre-event)	Composite clinical outcome (transfusion/interventions/signs/SMM)	PPH occurrence	Binary	Intrapartum / peripartum (during labor through delivery)
Holcroft (2024) [24]	Blood loss >500 mL within the first hour post-birth and/or need for blood transfusion	A) Anticipatory risk stratification (pre-event)	Documentation-derived phenotype (diagnosis/codes/chart review/labels)	PPH occurrence	Binary	Intrapartum / peripartum (during labor through delivery)
Hong (2025) [25]	Blood loss ≥1000 mL within 24 h postpartum OR red blood cell	A) Anticipatory risk	Composite clinical outcome (transfusio	PPH occurrence	Binary	Pre-delivery (antepartum / preoperative)

	transfusion ≥4 units after delivery	stratification (pre-event)	n/interventions/signs/SMM)			
Kovacheva (2025) [26]	Blood loss ≥1000 mL in the first 24 h after delivery	A) Anticipatory risk stratification (pre-event)	Blood-loss–based (EBL/QBL/quantified blood loss/lab proxy)	PPH occurrence	Binary	Labor admission / triage (pre-delivery baseline)
Krishnamoorthy (2022) [27]	PPH (postpartum hemorrhage; described as >500 mL blood loss within 24 h after vaginal birth)	A) Anticipatory risk stratification (pre-event)	N/A	PPH occurrence	Binary	N/A
Lengerich (2024) [28]	Clinician-diagnosed PPH with severe maternal morbidity (PPH+SMM); QBL not used	A) Anticipatory risk stratification (pre-event)	Composite clinical outcome (transfusion/interventions/signs/SMM)	Composite adverse outcome (PPH + transfusion/interventions/SMM)	Binary	Labor admission / triage (pre-delivery baseline)
Lérias-Cambeiro (2025) [29]	PPH defined as hemoglobin difference ≥2 g/dL between admission (T0) and 24 h postpartum (T1)	A) Anticipatory risk stratification (pre-event)	Blood-loss–based (EBL/QBL/quantified blood loss/lab proxy)	PPH occurrence	Binary	Labor admission / triage (pre-delivery baseline)
Li (2024) [30]	4-grade outcome (normal/mild/moderate/severe); thresholds not reported	B) Early warning / severity escalation (intrapartum or immediate postpartum)	Documentation-derived phenotype (diagnosis/codes/chart review/labels)	PPH severity / severe hemorrhage	Categorical	N/A
Li (2025a) [31]	Blood loss >1000 mL after cesarean section (WHO definition)	A) Anticipatory risk stratification (pre-event)	Blood-loss–based (EBL/QBL/quantified blood loss/lab proxy)	PPH occurrence	Binary	Pre-delivery (antepartum / preoperative)
Li (2025b) [32]	Normal <500 mL; Mild 500–1000 mL; Moderate 1000–1500 mL; Severe ≥1500 mL total blood loss	B) Early warning / severity escalation (intrapartum or immediate postpartum)	Blood-loss–based (EBL/QBL/quantified blood loss/lab proxy)	PPH severity / severe hemorrhage	Categorical	N/A

Liu (2022) [33]	PPH >500 mL (vaginal delivery); severe PPH ≥1000 mL	B) Early warning / severity escalation (intrapartum or immediate postpartum)	Blood-loss–based (EBL/QBL /quantified blood loss/lab proxy)	PPH occurrenc e	Binary	Intrapartum / peripartum (during labor through delivery)
Mehrnoush (2023) [34]	Recorded PPH (criteria not specified; background cites >500 mL vaginal or >1000 mL cesarean)	A) Anticipatory risk stratification (pre-event)	Documenta tion-derived phenotype (diagnosis/ codes/chart review/labe ls)	PPH occurrenc e	Binary	Intrapartum / peripartum (during labor through delivery)
Meyer (2024) [35]	PPH defined as quantitative blood loss (QBL) ≥1000 mL within 24 h after delivery	A) Anticipatory risk stratification (pre-event)	Blood-loss–based (EBL/QBL /quantified blood loss/lab proxy)	PPH occurrenc e	Binary	Labor admission / triage (pre-delivery baseline)
Raman (2025) [36]	N/A	A) Anticipatory risk stratification (pre-event)	N/A	PPH occurrenc e	Binary	N/A
Shah (2023) [37]	PPH outcome within 24 h after delivery; generally defined as EBL >500 mL (vaginal) or >1000 mL (cesarean), or blood transfusion within 24 h	A) Anticipatory risk stratification (pre-event)	Blood-loss–based (EBL/QBL /quantified blood loss/lab proxy)	PPH occurrenc e	Binary	Pre-delivery (antepartum / preoperative); Intrapartum / peripartum (during labor through delivery)
Song (2025) [38]	PPH defined as blood loss >500 mL within 24 h after vaginal delivery	A) Anticipatory risk stratification (pre-event)	Blood-loss–based (EBL/QBL /quantified blood loss/lab proxy)	PPH occurrenc e	Binary	Intrapartum / peripartum (during labor through delivery)
Susanu (2024) [39]	Intra-/postpartum hemorrhage assessed within first 24 h postpartum; PPH definition referenced ACOG/FIGO; severity classified per ATLS	A) Anticipatory risk stratification (pre-event)	Blood-loss–based (EBL/QBL /quantified blood loss/lab proxy)	PPH occurrenc e	Binary	N/A
Venkatesh (2020) [40]	PPH defined as estimated blood	A) Anticipatory risk	Blood-loss–based (EBL/QBL	PPH occurrenc e	Binary	Labor admission / triage (pre-

	loss (EBL) ≥1,000 mL	stratification (pre-event)	/quantified blood loss/lab proxy)			delivery baseline); Intrapartum / peripartum (during labor through delivery)
Wang (2024a) [41]	High hemorrhage group: blood loss ≥500 mL within 24 h after delivery; also states PPH screening thresholds: vaginal ≥500 mL, cesarean ≥1,000 mL	A) Anticipatory risk stratification (pre-event)	Documentation-derived phenotype (diagnosis/codes/chart review/labels)	PPH severity / severe hemorrhage	Binary	Intrapartum / peripartum (during labor through delivery)
Wang (2024b) [42]	PPH defined as blood loss exceeding 1000 mL within the first 24 hours after cesarean section	B) Early warning / severity escalation (intrapartum or immediate postpartum)	Blood-loss–based (EBL/QBL/quantified blood loss/lab proxy)	Quantitative blood loss (continuous; mL)	Continuous	Intrapartum / peripartum (during labor through delivery)
Wang (2025) [43]	PPH defined as blood loss ≥500 mL (vaginal) or ≥1000 mL (cesarean), within 2 hours after delivery	A) Anticipatory risk stratification (pre-event)	Documentation-derived phenotype (diagnosis/codes/chart review/labels)	PPH occurrence	Binary	Early postpartum (0–2 hours after delivery)
Westcott (2022) [44]	PPH defined as estimated blood loss ≥1000 mL at time of delivery	B) Early warning / severity escalation (intrapartum or immediate postpartum)	Blood-loss–based (EBL/QBL/quantified blood loss/lab proxy)	PPH occurrence	Binary	Intrapartum / peripartum (during labor through delivery)
Woo (2025) [45]	Two labels: (1) EBL–QBL: blood loss ≥500 mL (vaginal) or ≥1000 mL (cesarean); (2) cPPH: RBC transfusion ≥1 unit OR hysterectomy OR intrauterine tamponade balloon OR use of ≥3 medications (uterotonics or tranexamic acid)	A) Anticipatory risk stratification (pre-event)	Blood-loss–based (EBL/QBL/quantified blood loss/lab proxy)	PPH occurrence	Binary	Pre-delivery (antepartum / preoperative)
Xiao (2025) [46]	PPH within 24 h; blood loss >500 mL (vaginal) or	A) Anticipatory risk	Blood-loss–based (EBL/QBL/quantified	PPH occurrence	Binary	Pre-delivery (antepartum / preoperative)

	>1000 mL (cesarean)	stratification (pre-event)	blood loss/lab proxy)			
Yao (2025) [47]	PPH defined as blood loss ≥500 mL within 24 h after delivery	A) Anticipatory risk stratification (pre-event)	Blood-loss–based (EBL/QBL/quantified blood loss/lab proxy)	PPH occurrence	Binary	Intrapartum / peripartum (during labor through delivery)
Yuan (2023) [48]	High-risk PPH defined as postpartum blood loss >500 mL (also analyzed blood loss categories ≤200, 200–500, ≥500 mL)	B) Early warning / severity escalation (intrapartum or immediate postpartum)	Blood-loss–based (EBL/QBL/quantified blood loss/lab proxy)	PPH occurrence	Binary	Intrapartum / peripartum (during labor through delivery)
Zhang (2021) [49]	PPH within 24 h; labeled positive if blood loss >500 mL (vaginal) (paper also cites >1000 mL for cesarean generally)	C) Automated case identification / diagnosis (documentation-derived phenotyping)	Blood-loss–based (EBL/QBL/quantified blood loss/lab proxy)	PPH occurrence	Binary	Intrapartum / peripartum (during labor through delivery)
Zhang (2025) [50]	N/A (PPH definition not explicitly stated in Methods; PPH label derived from clinical records/EBL)	A) Anticipatory risk stratification (pre-event)	Blood-loss–based (EBL/QBL/quantified blood loss/lab proxy)	PPH occurrence	Binary	Pre-delivery (antepartum / preoperative)
Zorlu (2025) [51]	Blood loss ≥1000 mL within first 24h postpartum OR clinical signs of hypovolemia	A) Anticipatory risk stratification (pre-event)	Composite clinical outcome (transfusion/interventions/signs/SMM)	PPH occurrence	Binary	Pre-delivery (antepartum / preoperative); Intrapartum / peripartum (during labor through delivery)

Supplementary Material 6: Per-study Input Data Used for AI Modeling

Study	Data Source	Number of Participants	Number of Cases	Number of Controls	Mean Age	Median Age	Age Range	antepartum data	Intrapartum data	Demographics	Postpartum data
Ahmadzia (2024) [19]	Closed	185,413	5760	179653	N/A	27	11–58	Yes (prenatal/antepartum clinical features and labs)	Yes (labor/delivery factors incl. mode of delivery, oxytocin, tocolytics, anesthesia/hospital factors)	Yes (age, BMI, race/ethnicity, insurance, etc.)	No
Akazawa (2021) [20]	Closed	9894	188	9706	N/A	31	15–45	Yes	Yes	Yes	No
Alsentzer (2023) [21]	Closed	131284	2270	129014	N/A	N/A	N/A	Yes (from discharge summary text)	Yes (delivery mode, EBL, procedures)	Yes (age, race/ethnicity)	Yes (post-delivery events documented in discharge summary)
Ambeth Kumar (2022) [22]	Closed	2000	N/A	N/A	N/A	N/A	N/A	Yes	Yes	Yes	No
Dogru (2025) [23]	Closed	615	150	465	31.37	N/A	N/A	Yes	Yes	Yes	Yes
Holcroft (2024) [24]	Closed	430	108	322	N/A	N/A	N/A	Yes	Yes	Yes	No
Hong (2025) [25]	Closed	306	165	141	33.35	N/A	N/A	Yes	No	Yes	No
Kovacheva	Closed	87662	6,789	80,873	32.4	N/A	N/A	Yes	Yes	Yes	No

(2025) [26]											
Krishnamoorthy (2022) [27]	Closed	11000	1042	9958	N/A	N/A	N/A	No	No	No	No
Lengerich (2024) [28]	Closed	85766	641	85125	N/A	N/A	N/A	Yes	Yes	Yes	No
Lérias-Cambeiro (2025) [29]	Closed	147	35	112	31.4	N/A	N/A	Yes	Yes	Yes	No
Li (2024) [30]	Closed	68352	N/A	N/A	N/A	N/A	N/A	No	No	No	No
Li (2025a) [31]	Closed	845	236	609	N/A	N/A	N/A	Yes	Yes	Yes	No
Li (2025b) [32]	Closed	68352	4788	63564	N/A	N/A	N/A	No	No	No	No
Liu (2022) [33]	Closed	7821	850	6971	N/A	N/A	N/A	Yes	Yes	Yes	No
Mehrnoush (2023) [34]	Closed	8888	163	8725	N/A	N/A	N/A	Yes	Yes	Yes	No
Meyer (2024) [35]	Closed	5261	1321	3940	N/A	31	27-34	Yes	Yes	Yes	No
Raman (2025) [36]	Closed	N/A	N/A	N/A	N/A	N/A	N/A	No	No	No	No
Shah (2023) [37]	Closed	1576	40	1536	27.5	N/A	N/A	Yes	Yes	Yes	No
Song (2025) [38]	Closed	24833	1623	23210	N/A	29	26–33	Yes	Yes	Yes	Yes
Susanu (2024) [39]	Closed	203	68	135	28.8	N/A	N/A	Yes	Yes	Yes	No
Venkatesh	Closed	152279	7279	145000	N/A	N/A	N/A	Yes	Yes	Yes	No

(2020) [40]											
Wang (2024a) [41]	Closed	768	337	431	30.5	N/A	N/A	Yes	Yes	Yes	No
Wang (2024b) [42]	Closed	6144	N/A	N/A	N/A	N/A	N/A	Yes (maternal characteristics, comorbidities, labs)	Yes (intraoperative/anesthesia/surgical factors)	Yes (e.g., age)	No
Wang (2025) [43]	Closed	24110	663	23447	N/A	28	N/A	Yes (history, labs)	Yes (delivery-related factors)	Yes	Yes (postpartum medication included)
Westcott (2022) [44]	Closed	30867	2179	28688	32.7	N/A	N/A	Yes (history, labs, demographics)	Yes (vitals, medications, labor/delivery variables)	Yes	Yes (full “all data” model includes delivery outcomes)
Woo (2025) [45]	Closed	19992	EBL–QBL: 1465; cPPH: 748	EBL–QBL: 18527; cPPH: 19244	30	N/A	N/A	Yes (prenatal notes; obstetric history)	No	Yes	No
Xiao (2025) [46]	Closed	280	N/A	N/A	34	N/A	24–45	Yes	No	Yes	No
Yao (2025) [47]	Closed	1225	310	915	N/A	N/A	N/A	Yes	Yes	Yes	No
Yuan (2023) [48]	Closed	10520	N/A	N/A	N/A	N/A	N/A	Yes	Yes	Yes	No
Zhang (2021) [49]	Closed	3842	361	3481	N/A	N/A	N/A	Yes	Yes	Yes	No

Zhang (2025) [50]	Closed	581	258	323	33	N/A	N/A	Yes	No	Yes	No
Zorlu (2025) [51]	Closed	566	283	283	30.5	N/A	N/A	Yes	Yes	Yes	No

Supplementary Material 7: Per-study AI Model Characteristics

Study	Model Family	Specific architecture	AI Model task	Validation Type	Validation technique	Confusion Matrix
Ahmadzia (2024) [19]	Classical ML; Deep learning	Gradient Boosting; Logistic Regression; Multilayer Perceptron; Random Forest; SVM	Classification	Internal	Hold-out split	Yes
Akazawa (2021) [20]	Classical ML; Deep learning	Boosted Trees; Decision Tree; Logistic Regression; Neural Network; Random Forest; SVM	Classification	Internal	Hold-out split	No
Alsentzer (2023) [21]	LLM	Flan-T5	Classification	Internal	Hold-out split	No
Ambeth Kumar (2022) [22]	Deep learning	VGG; ZFNet	Classification	Internal	Hold-out split	No
Dogru (2025) [23]	Classical ML; Deep learning	Logistic Regression; Multilayer Perceptron; Random Forest; SVM	Classification	Internal	Hold-out split	No
Holcroft (2024) [24]	Classical ML	Random Forest	Classification	Internal	K-fold cross-validation	No
Hong (2025) [25]	Classical ML	LASSO; Logistic Regression	Classification	Internal	Hold-out split; K-fold cross-validation	No
Kovacheva (2025) [26]	Classical ML	LASSO; Logistic Regression; XGBoost	Classification	Internal + External	Hold-out split	No

Krishnamoorthy (2022) [27]	Classical ML; Deep learning	OBCSA; OSAE	Classification	Internal	K-fold cross-validation	Yes
Lengerich (2024) [28]	Classical ML	Explainable Boosting Machine	Classification	Internal + External	Hold-out split	No
Lérias-Cambeiro (2025) [29]	Classical ML	Ridge Logistic Regression	Classification	Internal	K-fold cross-validation	No
Li (2024) [30]	Classical ML	CatBoost	Classification	Internal	N/A	No
Li (2025a) [31]	Classical ML	Gradient Boosting Machine; Logistic Regression	Classification	Internal + External	Hold-out split	No
Li (2025b) [32]	Deep learning	Deep Neural Network; Logistic Regression; MWACGAN; Random Forest	Classification	Internal	Hold-out split	No
Liu (2022) [33]	Classical ML	KNN; LightGBM; Logistic Regression; Random Forest; XGBoost	Classification	Internal	Hold-out split; K-fold cross-validation	No
Mehrnoush (2023) [34]	Classical ML	Artificial Neural Network; Decision Tree; KNN; LightGBM; Logistic Regression; Random Forest; SVM; XGBoost	Classification	Internal	N/A	Yes
Meyer (2024) [35]	Classical ML	Gradient Boosting; LASSO; Logistic Regression; Random Forest	Classification	External	Hold-out split; K-fold cross-validation	No
Raman (2025) [36]	Classical ML	Decision Tree; Logistic Regression; Random Forest	Classification	N/A	N/A	No
Shah (2023) [37]	Classical ML	Decision Tree; GAIN; Logistic Regression; Naive Bayes; Random Forest		Internal	Hold-out split; K-fold cross-validation	No
Song (2025) [38]	Classical ML	CatBoost; KNN; LightGBM; Logistic Regression; Random Forest; SVM; XGBoost	Classification	Internal + External	Hold-out split; Temporal validation	No
Susanu (2024) [39]	Classical ML; Deep learning	Decision Tree; Naive Bayes; Neural Network;		Internal	N/A	No

		Random Forest; SVM				
Venkatesh (2020) [40]	Classical ML	LASSO; Logistic Regression; Random Forest; XGBoost	Classification	External	Temporal validation; K-fold cross-validation	No
Wang (2024a) [41]	Classical ML	AdaBoost; Random Forest	Classification	Internal	Hold-out split	Yes
Wang (2024b) [42]	Classical ML; Deep learning	Deep Neural Network; Random Forest Regression; XGBoost	Regression	Internal	Hold-out split	No
Wang (2025) [43]	Classical ML; Deep learning	Deep Neural Network	Classification	Internal	K-fold cross-validation	No
Westcott (2022) [44]	Classical ML	Gradient Boosting; Logistic Regression; Random Forest; SVM	Classification	Internal	Hold-out split	No
Woo (2025) [45]	LLM; Classical ML	Llama; Logistic Regression; XGBoost	Classification	Internal	Temporal validation	No
Xiao (2025) [46]	Deep learning	Logistic Regression; ResNet	Classification	Internal	Hold-out split	No
Yao (2025) [47]	Classical ML	CatBoost; LightGBM; Random Forest; SVM; XGBoost		Internal	Hold-out split; K-fold cross-validation	No
Yuan (2023) [48]	Classical ML	Random Forest	Classification	Internal	K-fold cross-validation	No
Zhang (2021) [49]	Classical ML	Ensemble; Naive Bayes; Neural Network; Random Forest; SVM; XGBoost	Classification	Internal	Hold-out split	No
Zhang (2025) [50]	Deep learning	LASSO; Logistic Regression; ResNet; Stacking Logistic Regression	Classification	External	Hold-out split	No
Zorlu (2025) [51]	Classical ML	Gradient Boosting; Logistic Regression; Naive Bayes; Random Forest; SVM	Classification	Internal	Hold-out split; K-fold cross-validation	No

Supplementary Material 8: Reviewers' judgments about each domain in "risk of bias" and "applicability concerns" for each included study

D1	Participants	+	Low risk
D2	Index test	?	Unclear
D3	Reference standard	-	High risk
D4	Analysis		

Risk of Bias					
#	Study Ref	**D1**	**D2**	**D3**	**D4**
1	Ahmadzia 2024	-	+	+	+
2	Akazawa 2021	-	+	+	+
3	Alsentzer 2023	-	+	+	+
4	Ambeth Kumar 2022	?	+	+	+
5	Dogru 2025	+	+	+	+
6	Holcroft 2024	+	+	+	+
7	Hong 2025	+	+	+	+
8	Kovacheva 2025	-	+	+	+
9	Krishnamoorthy 2022	-	+	+	+
10	Lengerich 2024	-	+	+	+
11	Lérias-Cambeiro 2025	+	+	+	+
12	Li 2024	?	+	+	+
13	Li 2025a	+	+	+	+

14	Li 2025b	-	+	+	-
15	Liu 2022	-	+	+	+
16	Mehrnoush 2023	-	+	+	+
17	Meyer 2024	+	+	+	+
18	Raman 2025	?	+	+	-
19	Shah 2023	-	+	+	+
20	Song 2025	-	+	+	+
21	Susanu 2024	+	+	+	+
22	Venkatesh 2020	-	+	+	-
23	Wang 2024a	+	+	+	+
24	Wang 2024b	?	+	+	+
25	Wang 2025	-	+	?	+
26	Westcott 2022	-	+	+	+
27	Woo 2025	?	+	+	+
28	Xiao 2025	?	+	+	+
29	Yao 2025	-	+	+	+
30	Yuan 2023	?	+	+	-
31	Zhang 2021	-	+	+	+
32	Zhang 2025	+	+	?	+
33	Zorlu 2025	+	+	+	?

Applicability Concerns				
#	**Study Ref**	**D1**	**D2**	**D3**
1	Ahmadzia 2024	-	+	+
2	Akazawa 2021	-	+	+
3	Alsentzer 2023	-	+	+
4	Ambeth Kumar 2022	?	+	+
5	Dogru 2025	+	+	+
6	Holcroft 2024	+	+	+
7	Hong 2025	+	+	+
8	Kovacheva 2025	-	+	+
9	Krishnamoorthy 2022	+	+	+
10	Lengerich 2024	-	+	+
11	Lérias-Cambeiro 2025	+	+	+
12	Li 2024	?	+	+
13	Li 2025a	+	+	+
14	Li 2025b	-	+	+
15	Liu 2022	+	+	+
16	Mehrnoush 2023	-	+	+
17	Meyer 2024	+	+	+
18	Raman 2025	?	+	+
19	Shah 2023	-	+	+

20	Song 2025	-	+	+
21	Susanu 2024	+	+	+
22	Venkatesh 2020	-	+	+
23	Wang 2024a	+	+	+
24	Wang 2024b	?	+	+
25	Wang 2025	-	+	?
26	Westcott 2022	+	+	+
27	Woo 2025	?	+	+
28	Xiao 2025	?	+	+
29	Yao 2025	-	+	+
30	Yuan 2023	?	+	+
31	Zhang 2021	+	+	+
32	Zhang 2025	+	+	?
33	Zorlu 2025	+	+	+
